# Quantitative prediction of intravenous drug interactions caused by cytochromes P450 inhibitors and inducers

**DOI:** 10.1002/bcp.70548

**Published:** 2026-04-04

**Authors:** Vianney Tuloup, Michel Tod, Laurent Bourguignon

**Affiliations:** ^1^ Pharmacy Department CHRU Tours Tours France; ^2^ Pharmacy Faculty Tours University Tours France; ^3^ Inserm 1327 ISCHEMIA Tours University Tours France; ^4^ Pharmacology‐Physiology‐Toxicology Department, ISPB Pharmacy Faculty of Lyon University of Lyon Lyon France; ^5^ Laboratoire de Biométrie et Biologie Évolutive Univ Lyon Université Claude Bernard Lyon 1, UMR CNRS 5558 Villeurbanne France; ^6^ Pharmacy Department University Hospital of Lyon GH Nord Lyon France

**Keywords:** cytochrome P450, drug interactions, modelling and simulation, pharmacometrics

## Abstract

**Aims:**

Pharmacokinetic interaction studies typically focus on oral administration, but intravenous (IV) administration bypasses intestinal degradation and hepatic first‐pass metabolism, leading to distinct drug–drug interaction (DDI) magnitude. This study aimed to develop a predictive model for DDIs involving IV‐administered drugs.

**Methods:**

Drugs metabolized by the five major cytochrome P450 enzymes were analysed. Data from IV DDI studies, including area under the concentration‐time curve (AUC) ratios of object drugs with and without precipitants, were collected. A Bayesian approach estimated IV contribution ratios (CR) from published oral CR values. Predictions were validated by assessing whether predicted‐to‐observed ratios fell within 50–200% of observed values.

**Results:**

Data for 33 drugs and 87 AUC ratios involving 26 precipitants were analysed. In the training set, 95% of AUC ratios were within the acceptability range, with a mean bias of 0.09 mg·h/L and 16% imprecision. In the validation set, 85% of predictions were within range, with a bias of −0.012 mg·h/L and 27.3% imprecision. Mean AUC ratios for inhibitors were 2.28 (oral) and 1.78 (IV). For inducers, mean AUC ratios were 0.209 (oral) and 0.62 (IV).

**Conclusions:**

Despite the limited dataset, the model demonstrated robust performance with 85% of predictions validated. It expands the DDI‐predictor framework for quantitative prediction of cytochrome‐mediated drug–drug interactions affecting drug exposure to intravenously administered object drugs, thereby providing a tool to predict and understand IV‐specific DDIs effectively.

**What is already known about this subject**

Drug interactions are well‐described for oral administration. Intravenous administration of an object drug bypasses the gastro‐duodenal metabolism and therefore may decrease the magnitude of the interaction.

**What this study adds**

We propose a general framework for drug–drug interactions following intravenously administrated object drug. Cytochrome contribution ratios were defined without first‐pass hepatic metabolism. Because intravenous administration results in weaker interactions, dosage adjustments are required but typically less significant than for oral administration.

## 1 INTRODUCTION

Human cytochromes P450 (CYP) enzymes have a major role in the metabolism of endogenous and exogenous compounds. Among more than 50 cytochromes, 5 major CYP accounts for more than 90% of the metabolism activity of this enzyme class, the most important one being CYP450 3A4.^1^, ^2^, ^3^ CYP are involved in the metabolism of more than 50% of drugs on the market^4^ and are also the target of many precipitants (inducer or inhibitor compounds), leading to potential meaningful pharmacokinetic drug–drug interaction (DDI).^5^ Those interactions may lead to inefficacy due to a decrease exposure^6^ or toxicity induction by a clearance decrease^7^ in case of co‐administration of object and precipitant. Moreover, due to their ubiquitarian presence in the organism, cytochromes are likely to cause DDI for both oral and parenteral pathways, and it has been clearly documented that DDI magnitude is influenced by the object drug administration pathway.^8^ A major example is intestinal and gut‐wall absorption, which takes a major part in bioavailability and is highly susceptible to DDI when a drug is administered by oral route but not in IV infusion. Numerous studies tried to evaluate the prevalence of cytochrome in the gut‐wall and intestine, and to quantify the part of intestinal metabolism in total metabolism with the help of Physiologically‐Based Pharmacokinetic models.^9^, ^10^

However, most pharmacokinetic interaction studies are evaluated with oral intake of the object and the precipitant. Those studies do not consider the specificity of intravenous administration (IV), such as the lack of intestinal absorption or first‐pass metabolism. Therefore, we built an In Vivo semi‐mechanistic static model (IMSM) able to predict the hepatic contribution of the five major cytochromes in the metabolism of intravenously administered drugs, and to estimate the impact on drug exposure of interactions between intravenous objects and per os/IV precipitants, as previously published for oral/oral DDI.^11^, ^12^

## 2 METHODS

We derived the IMSMs built by Ohno, Tod, Goutelle et al.,^11^, ^12^, ^13^, ^14^, ^15^, ^16^ based on per os administration of drugs, to model the interaction between intravenous object and per os/IV precipitant.

The general metric used to quantify the interaction magnitude is the ratio of the area under the concentration‐time curve (AUC) of the object in the presence of a precipitant (AUC*) and the object administered alone (AUC), as shown in Equations 1 and 2.

(eq 1) $$\frac{{\mathrm{AUC}}^{*}}{\mathrm{AUC}}=\frac{1}{\left(1-{\mathrm{CR}}_{\mathrm{cyp}}*{\mathrm{IR}}_{\mathrm{cyp}}\right)}$$

in case of inhibition

(eq 2) $$\frac{{\mathrm{AUC}}^{*}}{\mathrm{AUC}}=\frac{1}{\left(1+{\mathrm{CR}}_{\mathrm{cyp}}*{\mathrm{IC}}_{\mathrm{cyp}}\right)}$$

in case of induction

where CRcyp is the contribution ratio (fraction of the object drug's clearance due to metabolism by a specific CYP enzyme, which may vary between 0 and 1), IR the inhibition ratio of the inhibitor, reflecting the potency of CYP inhibition and IC the induction ratio of the inducer, reflecting the potency of CYP induction by the inducer.

*IR* varies between −1 and 0, resulting in an AUC ratio oscillating between 1 and a positive infinite value. An AUC ratio equal to 1 accounts for a lack of interaction in case of inhibition.

*IC* may vary between 0 and a positive theoretical infinite value. However, most known inducers have an *IC* value below 17. The corresponding AUC ratio oscillates between 1 (no interaction) and 0 (maximal induction).

*CR* and *IR‐IC* were defined previously for orally administered drugs. The main objective of this study was to introduce intravenous drugs into our frameworks and estimate new contribution ratios involving hepatic metabolism without gut wall cytochrome activity to predict drug interaction between an intravenous object and an IV or per os precipitant, using data from the literature.

We first obtained values for interaction potency using previously published data. We used the Bayesian regression method to estimate the contribution ratio of any substrate available. When multiple studies were available for an object–precipitant couple, one study was used for estimation of *CR*, and others were set apart for a validation dataset.

### 2.1 Initial estimation from literature data

A literature search was performed to retrieve studies that evaluated drug interaction between IV‐IV object and precipitant or IV‐per os object and precipitant. Main keywords used were ‘pharmacokinetic’, ‘drug interaction’, ‘intravenous or IV’ and the name of each drug present in the DDI‐predictor database^17^ (available at www.ddi-predictor.org). Some data were also extracted from summaries of product characteristics from the Food and Drug Agency or the European Medicines Agency. Studies were included if the object was administered intravenously and if information on dosage regimen, AUC or drug clearance was available for the object alone and the object plus precipitant. Only studies undertaken in humans were considered.

Intravenous contribution ratios for each CYP involved were calculated by rearrangement of Equations 1 and 2, as follows in Equation 3 and therefore added in our Bayesian estimation of model parameters.

(eq 3) CR=AUC*AUC−1AUC*AUC*IR

We used IR values previously calculated for the oral pathway as an initial estimate of intravenous CR values.^12^, ^13^, ^14^, ^16^, ^18^ When several studies were found on the same object, we used those with multiple administrations of the precipitant to truly reflect the magnitude of interaction, especially in the case of induction, which may take several days to attain stability. Unused studies were preserved for the external validation step.

### 2.2 Bayesian estimation of model parameters

Bayesian estimation implemented in runjags (Matthew J. Denwood (2016), Journal of Statistical Software, 71(9)) package using R Statistical Software (v4.1.2; R Core Team 2021) was used to obtain the final estimation of CR values in case of intravenous administration. This approach was possible as we had previously published information on the oral contribution ratio and the revised CR obtained from Equation 3. The JAGS software provided the Bayesian posterior distribution of the CR parameter and confidence interval based on the model and the prior data implemented.

For the j‐th object and i‐th inhibitor, the predicted AUC ratio was encoded as described in Equation 4

(eq.4) $${\text{pred}}_{\mathrm{ij}}=\frac{1}{1-\left(\sum {\mathrm{CR}}_{j}{\mathrm{IR}}_{i}\right)}$$

$${\text{AUCratio}}_{\mathrm{ij}}\sim N\;\left({\text{pred}}_{\mathrm{ij}},\text{tauAUC}\right)$$

Where *pred*_*ij*_ and *AUC ratio*_*ij*_ are the predicted and observed AUC ratio of each cytochrome and inhibitor couple (*CRj, IRi*), respectively, and *CRj* is the Bayesian posterior estimation of the j‐cytochrome contribution ratio.

The parameter *tauAUC* represents the precision (i.e., the reciprocal of the variance) of the AUC ratio distribution. AUC ratios and contribution ratios (CRs) were assumed to follow normal distributions. The precision parameters of these distributions, denoted *tau*, were assigned gamma prior distributions as follows: tau.CR ~ G (4,1), *tau. AUC* ~G (0.1,2.5) for CR and AUC ratio distributions, respectively. Gamma distributions were used as common priors for the precision of normal distributions, ensuring strictly positive values.^12^, ^19^

The mean of a gamma distribution is defined as α/β. Accordingly, the prior mean of the precision parameters *tau* was 4 for CRs and IRs and 0.04 for AUC ratios. These values correspond to expected variances of 0.25 and 25, and expected standard deviations of 0.5 and 5 for CRs/IRs and AUC ratios, respectively. These precision priors control variability around deterministic prior means of CRs, IRs and AUC ratios derived from Equations 3 and 4.

Posterior distributions of AUC ratios, CRs and IRs were obtained using Markov chain Monte Carlo simulations implemented in *runjags*. Convergence was assessed by evaluating the stability of posterior distributions, and goodness‐of‐fit was assessed by visual inspection of residual scatter plots. Posterior distributions were examined for multimodality, which would indicate a conflict between the specified prior distributions and the observed data.

### 2.3 External validation

AUC ratios predicted in the first step were compared with observed data of a validation dataset not used during the modelling step. Ratios included in a 50–200% range of observed values were deemed acceptable, as in our previous works.^11^, ^12^, ^15^ Bias and imprecision were calculated as the median of prediction errors and the median of absolute prediction errors, respectively.

Nomenclature of Targets and Ligands Key protein targets and ligands in this article are hyperlinked to corresponding entries in http://www.guidetopharmacology.org, and are permanently archived in the Concise Guide to PHARMACOLOGY 2021/22 (Alexander et al., 2021).^20^, ^21^

## 3 RESULTS

From the 224 object drugs listed in the ddi‐predictor database, 28 had exploitable data on IV administration with a per os precipitant, and five objects had available studies on IV‐IV interaction studies. This review results in an 87 AUC ratio reported from the literature, with 4 inducers and 22 inhibitors. Initial AUC estimations with observation references are reported in Tables 1 and 2, for IV‐ per os and IV‐IV interaction, respectively.

**TABLE 1:** : Summary of published studies with IV object and oral precipitant used for model construction. *AUC: area under the curve*. AUC*: area under the curve of the object in case of precipitant co‐administration.

Object name	Object AUC (ng.h/mL)	Precipitant name (	Precipitant dose (mg)	AUC* (ng.h/mL)	AUCratio	Ref
Alfentanil	98.2	erythromycin	600	132.07	1.345	^22^
alfentanil	117.8	fluconazole	75	231.5	1.965	^23^
alfentanil	59	GFJ	2 oz	57	0.966	^24^
alfentanil	59	ketoconazole	1000	291	4.932	^24^
alfentanil	59	rifampicin	400	21	0.356	^24^
alfentanil	97	voriconazole	1200	353	3.639	^25^
Antipyrine	337.2	ciprofloxacine	1000	522.8	1.550	^26^
Caffeine	224	ketoconazole	400	267	1.192	^27^
Caffeine	224	terbinafine	500	290	1.295	^27^
Ciclosporin	10.09	rifampicin	600	7.293	0.723	^28^
cyclophosphamide	247	aprepitant	80	317	1.283	^29^
Dexamethasone	246	itraconazole	200	796	3.236	^30^
Diazepam	9.78	cimetidine	300	11.76	1.202	^31^
Diazepam	4.63	ciprofloxacine	300	6.97	1.505	^32^
Diazepam	196	omeprazole	20	266	1.357	^33^
Diazepam	43.3	sertraline	1500	46.95	1.084	^34^
Diazepam	158	valproic acid	1000	219	1.386	^35^
Digoxine	22.1	rifampicin	600	20	0.905	^36^
Docetaxel	3035	St Jonh's Wort	900	2682	0.884	^37^
Duloxetine	21	fluvoxamine	100	56.6	2.695	^38^
Felodipine	82	GFJ	1	74.8	0.912	^39^
Fentanyl	6.1	fluconazole	200	7.7	1.262	^40^
Fentanyl	5.1	ketoconazole	200	6.8	1.333	^41^
Fentanyl	4.8	ritonavir	800	8.8	1.833	^42^
Fentanyl	6.1	voriconazole	400	8.5	1.393	^40^
Lidocaine	111.7	amiodarone	500	131.7	1.179	^43^
Lidocaine	99.2	cimetidine	1200	131.94	1.330	^44^
Lidocaine	1926	ciprofloxacine	500	2444	1.269	^45^
Lidocaine	1902	erythromycin	1500	2105	1.107	^46^
Lidocaine	1939	fluvoxamine	100	3315	1.710	^47^
Lidocaine	1902	itraconazole	200	2211	1.162	^46^
Lidocaine	6.14	omeprazole	40	6.67	1.086	^48^
Lidocaine	76.3	propafenone	675	81.7	1.071	^49^
Lidocaine	111.7	ranitidine	300	107.14	0.959	^50^
Methylprednisolone	6822	aprepitant	125	9122	1.337	^51^
Methylprednisolone	871	diltiazem	180	1299	1.491	^52^
Methylprednisolone	698	itraconazole	200	1698	2.433	^53^
Methylprednisolone	829	ketoconazole	200	1953	2.356	^54^
Mexiletine	7.63	cimetidine	800	7.04	0.923	^55^
Mexiletine	7.63	ranitidine	600	7.06	0.925	^55^
Midazolam	75.4	aprepitant	125	112.4	1.491	^56^
Midazolam	216.3	diltiazem	240	372	1.720	^57^
Midazolam	70.2	ketoconazole	200	354	5.043	^58^
Midazolam	11.6	posaconazole	400	51.3	4.422	^59^
Midazolam	40.9	rifampicin	600	24.1	0.449	^60^
Midazolam	103.3	St Jonh's Wort	900	81.2	0.786	^61^
Midazolam	73.8	telithromycin	800	159	2.154	^62^
Midazolam	151	voriconazole	800	534	3.536	^7^
Midazolam	115.7	ticagrelor	360	103.7	0.896	^63^
Moclobemide	2.146	cimetidine	800	3.534	1.647	^64^
Nifedipine	38.1	rifampicin	600	26.7	0.701	^65^
Nisoldipine	7.492	cimetidine	800	7.62	1.017	^66^
Ondansetron	1268	aprepitant	600	1456	1.148	^67^
Ondansetron	326	rifampicin	375	170	0.521	^68^
Oxycodone	121.7	rifampicin	600	55	0.452	^69^
Phenytoin	245	amiodarone	200	342	1.396	^70^
Prednisolone	2.373	ketoconazole	200	3.256	1.372	^71^
Quinidine	2251.6.7	rifampicin	600	606.7	0.269	^72^
Ropivacaine	112.99	fluvoxamine	200	357.14	3.161	^73^
Ropivacaine	113.0	ketoconazole	50	132.45	1.172	^73^
Tacrolimus	653.9	rifampicin	600	427	0.653	^74^
Theophylline	96.11	ciprofloxacine	1500	121.74	1.267	^75^
Theophylline	6.52	diltiazem	180	8.23	1.263	^76^
Theophylline	291	erythromycin	1000	308	1.058	^77^
Theophylline	8.47	verapamil	360	10.61	1.252	^78^
Tolbutamide	56.5	sertraline	200	67.11	1.188	^79^

**TABLE 2:** Summary of studies with both intravenous object and precipitant used for model construction. *AUC*: *area under the curve*.

Object	Precipitant	Precipitant dose (mg)	AUC ratio	Ref
alfentanil	diltiazem	169	1.24	^80^
alfentanil	fluconazole	400	2.07	^23^
Ceftriaxone	azithromycine	500	1.00	^81^
Doxorubicine	ciclosporine	60	1.55	^82^
Doxorubicine	ciclosporine	12.98	1.81	^83^
Etoposide	ciclosporine	15	1.80	^84^
Etoposide	ciclosporine	4.5	1.59	^85^
Midazolam	diltiazem	169	1.24	^80^
Theophylline	cimetidine	1200	0.92	^86^
Theophylline	cimetidine	1200	1.13	^87^
Theophylline	cimetidine	1200	1.50	^88^

As we do not expect a change in inhibition ratio or induction potency regarding the administration pathway, IR and IC previously described for oral administration were used in our model. However, we reconsidered the induction potency of rifampicin 1200 mg per day, as predicted values of the AUC ratio with IV object with oral rifampicin co‐administration greatly overestimated the effect of induction. Subsequently, a Bayesian estimation for *IC* rifampicin was modelled, using the published studies of rifampicin interaction in the construction model dataset: *IC* value for oral induction after 1200 mg/day of rifampicin was previously estimated at 7.7 for CYP450 3A4, and Bayesian estimation reconsidered it to 3.35 +/− 3.8 (mean and standard deviation).

**FIGURE 1:**
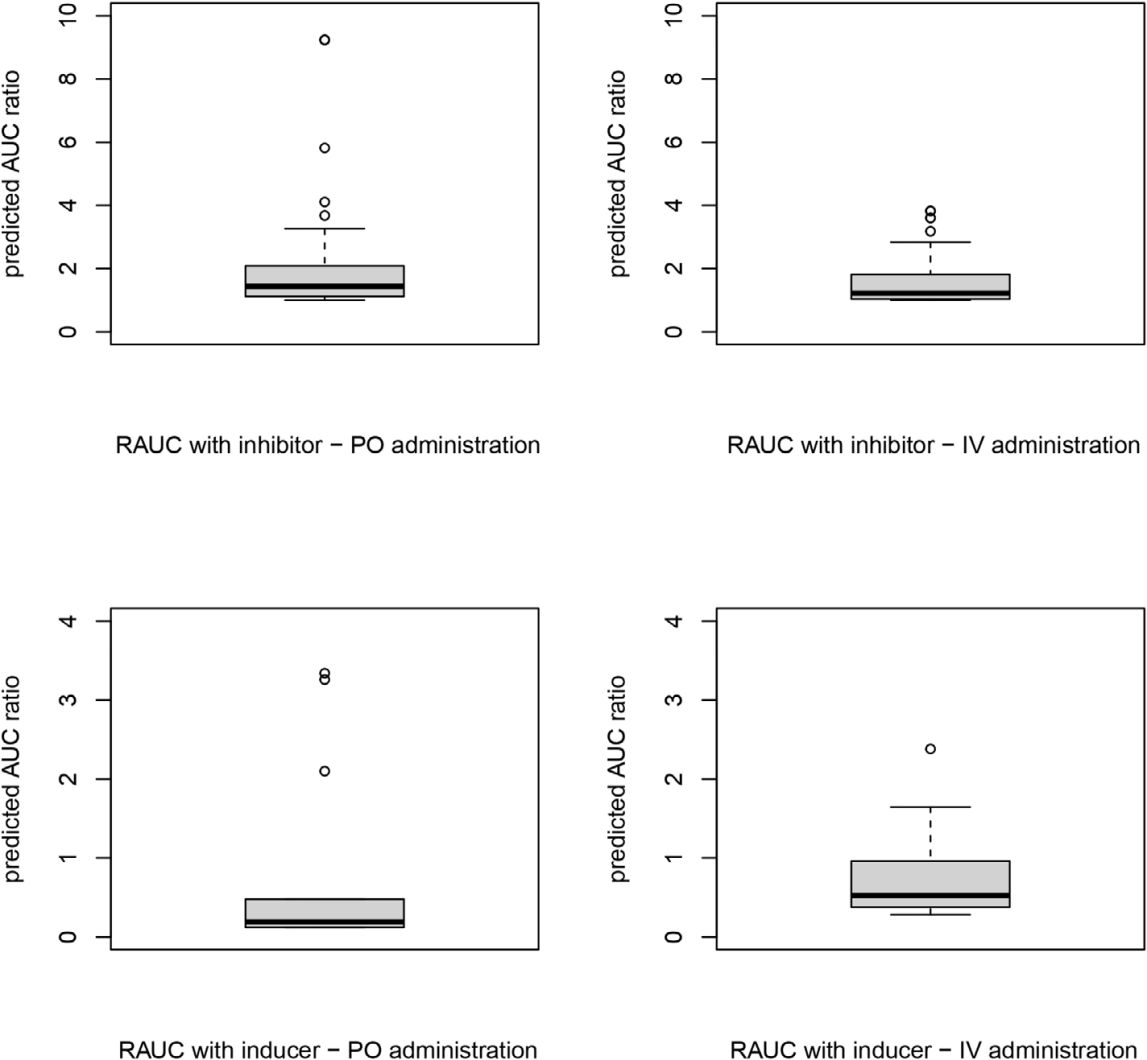
Boxplot of the AUC ratios according to the four possible scenarios: respectively PO administration and inhibitor, IV administration and inducer, PO administration and inducer and IV administration and inducer. AUC: area under the concentration time curve.

Using the estimated contribution ratio (as shown in Table 3), AUC ratios were calculated and compared to the observed AUC ratio of the literature provided in Figure 1. For inhibition, the mean AUC ratio when objects were administered by oral route was 2.28 (+/− 2.0) while 1.78 (+/− 1.64) for IV administration. Mean AUC ratio for oral administration of objects with an inducer was 0.209 (+/− 0.14) while 0.62 (+/− 0.19) for IV object administration. A summary table of the induction and inhibition ratios used in our study is provided in Supplementary Material 1.

**TABLE 3:** : Bayesian estimation of contribution ratio in case of intravenous administration. E: hepatic extraction ratio. L stands for low, M for moderate and H for high extraction ratio.

Object	E	CR 3A4	CR 2D6	CR2C9	CR2C12	CR1A2
Alfentanil	M	0.74	0	0	0	0
Antipyrine	L	0.09	0	0	0.003	0.17
Caffeine	L	0.0	0	0	0	0.707
Ciclosporin	M	0.46	0	0	0	0
Cyclophosphamide	L	0.13	0	0.290	0.302	0
Docetaxel	L	0.15	0	0	0	0
Dexamethasone	L	0.67	0	0	0	0
Diazepam	L	0.01	0	0	0.410	0
Digoxine	L	0.02	0.010	0.012	0.016	0.012
Midazolam	M	0.75	0	0	0	0
Methylprednisolone	L	0.51	0	0	0	0
Fentanyl	H	0.24	0	0	0	0
Lidocaine	M	0.16	0	0	0	0.250
Nifedipine	M	0.52	0	0	0	0
Mexiletine	L	0.0	0.083	0	0	0.021
Felodipine	M	0.76	0	0	0	0
Duloxetine	M	0.0	0.156	0	0	0.482
Moclobemide	M	0.0	0	0	0.502	0
Nisoldipine	M	0.64	0	0	0	0
Ondansetron	M	0.39	0	0	0	0
Oxycodone	M	0.33	0.039	0	0	0
Ropivacaine	M	0.18	0	0	0	0.558
Quinidine	M	0.25	0	0	0	0
Phenytoin	L	0.0	0	0.511	0.038	0
Prednisolone	L	0.04	0	0	0	0
Theophylline	L	0.0	0	0	0	0.323
Tacrolimus	L	0.81	0	0	0	0

**FIGURE 2:**
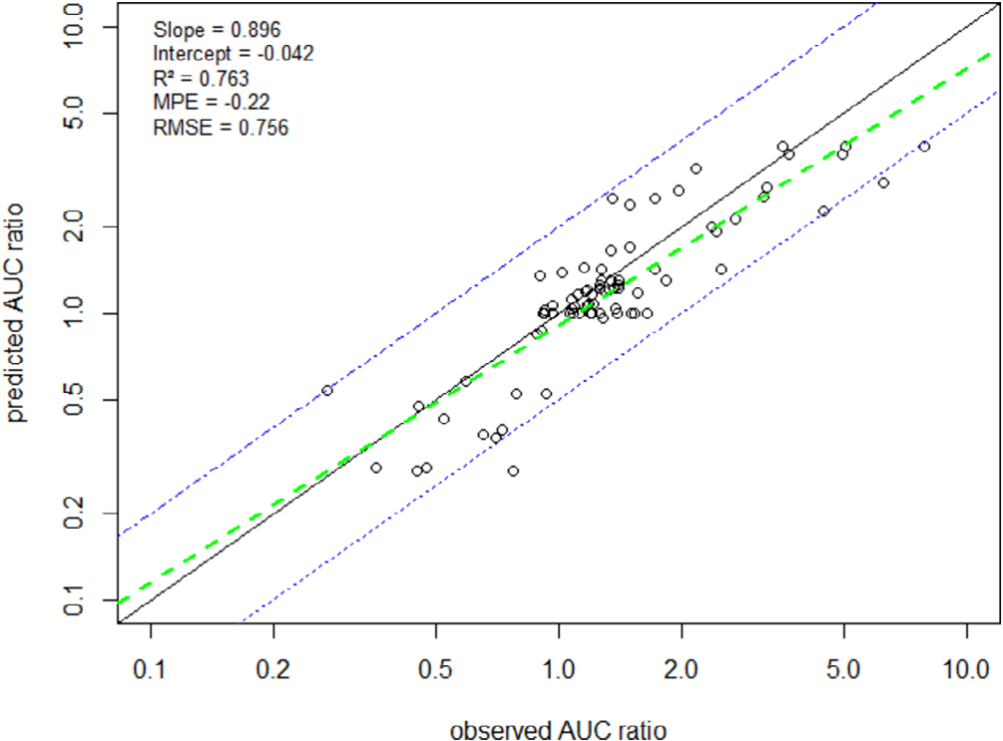
Observed *vs.* predicted AUC ratios for interactions between intravenously administered object and orally administered precipitants in the construction dataset. AUC: area under the concentration–time curve; MPE: mean prediction error; RMSE: root mean squared error. The solid line represents the identity line; the blue dashed lines indicate the 0.5– to 2‐fold interval, and the green dashed line represents the regression slope.

A graph of observed *vs*. predicted AUC ratios for the construction dataset is available in Figure 2 below. Bias and imprecision were respectively −0.095 mg.h.^L‐1^ and 16.6%.

**FIGURE 3:**
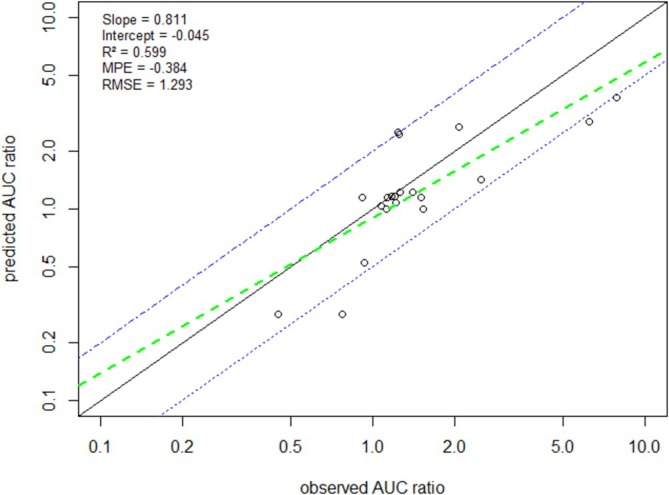
Observed *vs.* predicted AUC ratio for an external validation dataset. AUC: area under the concentration time curve;; MPE: mean prediction error; RMSE: root mean squared error. The solid line represents the identity line; the blue dashed lines indicate the 0.5– to 2‐fold interval, and the green dashed line represents the regression slope.

Figure 3 below represents external validation with the testing dataset. Bias for this testing dataset was at −0.112 mg.h/L and imprecision at 27.3%.

We performed a comparison of AUC ratios according to the hepatic extraction coefficient (E). For drugs with moderate hepatic extraction (0.3 < E < 0.7), the median AUC ratio reported in the literature following intravenous administration was 1.70 ± 1.71 (range: 0.27–7.82; median: 1.11), compared with 2.33 ± 2.65 (range: 0.12–9.24; median: 1.25) for oral administration. For drugs with low hepatic extraction (E < 0.3), the mean AUC ratio was 1.41 ± 0.53 (range: 0.65–3.24; median: 1.32) for intravenous administration and 1.56 ± 0.72 (range: 0.12–3.26; median: 1.44) for oral administration.

## 4 DISCUSSION

In this study, we extended the IMSM approach built by Ohno, Tod et al^11^, ^12^, ^16^ to intravenously administered object drugs. Similar to previous work on CYP1A2,^16^ Bayesian estimation was used to redefine, for each drug, the contribution ratio of the five major cytochromes to take into account the absence of intestinal metabolization and first‐pass metabolic effect. Using literature data on DDI with intravenous objects and IV or oral precipitants, our approach showed a good predictive performance, with a limited bias and an acceptable precision of predicted AUC ratio, when compared to observed values in clinical studies. Moreover, all values but three of the predicted *vs*. observed plot are in the predefined acceptability range of 0.5–2‐fold of observed value. All values concerned a midazolam injection, one with rifampicin and two with inhibitors (ketoconazole and posaconazole).

To allow reliable predictions, the induction potency of rifampicin 1200 mg per day was re‐estimated, from 7.7 to 3.35. This decrease in the induction potency of rifampicin for intravenous objects was observed in multiple cases, as for nifedipine,^65^ tacrolimus^74^ or midazolam^6^ and indicates a major implication of gut wall enzymes for those drugs.^89^, ^90^ Watkins and Wacher, among others, worked on the repartition of enzymatic activity of cytochrome 3A4, and concluded a 70/30 ratio of activity in the gut and liver,^91^, ^92^ which supports our findings. Asaumi et al found that intestinal effects of P‐gp–mediated DDIs had their greatest impact on the pharmacokinetics of digoxin, with a minimal impact on the liver and kidney,^93^ comforting our decision of rifampicin IX lowering in case of Iv administration.

We observed a decrease of contribution ratio between IV et per os administration for each drug studied, in coherence with the literature. Since intravenous infusion bypasses intestinal enzymatic activity and hepatic first‐pass metabolism, the average amount of cytochrome involved in DDI is lower compared to oral administration, thereby reducing the potential magnitude of DDI.

Magnitude of CR decrease may vary between objects. One first explanation could be the affinity of those objects for efflux intestinal transporters such as P‐glycoprotein, which can increase the gastrointestinal residence time and the enzymatic cytochrome activity on objects. This is relevant and described for drugs such as tacrolimus, alfentanil, felodipine or nisoldipine. Qian et al, in 2019, developed a semi‐physiologically based pharmacokinetic model designed for CYP3A and P‐gp objects drugs with rifampicin.^94^ Their conclusions regarding the influence of posology on rifampicin induction potency (IC) and the reduced impact of DDI observed with IV administration align with our findings, as we demonstrated across the five major cytochromes. However, contrary to PBPK modelling, in the IMSM approach contribution ratio (CR) for oral administration describes not only the metabolism (hepatic, digestive) but also the effect of digestive efflux transporters such as P‐gp. The estimation of *CR* when objects are administered by the intravenous route made it possible to overcome the P‐gp influence and determine a more specific contribution of hepatic metabolic activity. This may explain the need for rifampicin induction potency re‐estimation as it is one of the strongest P‐gp inducer.

The impact of drug–drug interactions is strongly influenced by both the hepatic extraction ratio and the route of administration. For drugs with moderate hepatic extraction, oral administration tends to amplify the magnitude of interactions due to first‐pass metabolism, whereas intravenous administration mitigates this effect. Interestingly, some drugs with low hepatic extraction, such as tacrolimus and phenytoin, are nonetheless extensively metabolized. In these cases, intravenous administration can substantially reduce interactions with potent inhibitors or inducers, despite a low extraction ratio. These observations underscore the importance of considering both metabolic capacity and route of administration when predicting clinically relevant interactions, rather than relying solely on hepatic extraction alone.

Our study had some limitations. First, a limited number of drug interaction studies have been published with IV object and oral or IV precipitant. Therefore, external validation was done with a limited dataset. Moreover, this paucity of data implies a lack of information on some objects, with data relying only on one DDI pharmacokinetic study to assess the influence of precipitants on drug exposure. Only contribution ratios were re‐estimated with oral prior information. Further studies are needed to exploit those results not only on objects but also on intravenous precipitants. This may help clinicians to adjust low‐margin therapeutic doses in case of modification of the administration route.

## 5 CONCLUSION

We developed here an extension of the IMSM approach used in DDI management, in order to predict the magnitude of DDI when an object drug is administered by IV route. For 28 different drugs, an estimation of the contribution ratios of 5 major CYPs when the drug is administered IV was undertaken. Contrary to previous studies based on global ‘whole‐body’, the *CRs* calculated in this study are not biased by hepatic first‐pass and intestinal metabolism. Calculated *CR* are consistent with the literature, and predicted AUC ratios are in the usually accepted range of 0.5–2‐fold of observed values. This extension of the DDI‐predictor tool is expected to be available for pharmacists and prescribers, as a convenient tool to avoid drug–drug interaction and reduce adverse effect.

## AUTHOR CONTRIBUTIONS

All authors contributed to the study conception and design. Data collection and analysis were performed by Vianney Tuloup and Laurent Bourguignon. The first draft was written by Vianney Tuloup and all authors commented on previous versions of the manuscript. All authors read and approved the final manuscript.

## CONFLICT OF INTEREST STATEMENT

VT, MT and LB have contributed to the DDI‐predictor.org website, which is a free webtool, without any profit for the authors. The authors have no conflicts of interest that are relevant to the content of this study.

## CODE AVAILABILITY

The code generated during the current study is available from the corresponding author on reasonable request.

## Supporting information


**Table S1.** Induction Potency and Inhibition Ratio of all perpetrators used during our study. All values are given for oral intake. *IX: induction or inhibition ratio. The inhibition ratio refers to values between −1 and 0, while the induction ratio refers to values greater than 0*.

## Data Availability

The datasets generated during and/or analysed during the current study are available from the corresponding author on reasonable request.

## References

[1] Slaughter RL , Edwards DJ . Recent advances: the cytochrome P450 enzymes. Ann Pharmacother SAGE Publications Inc. 1995;29(6):619‐624. doi:10.1177/106002809502900612 7663035

[2] Lynch T , Neff AP . The effect of cytochrome P450 metabolism on drug response, interactions, and adverse effects. Am Fam Physician. 2007;76:391‐396. 17708140

[3] Wilkinson GR . Drug metabolism and variability among patients in drug response. N Engl J Med. Massachusetts Medical Society. 2009;353(21). doi:10.1056/NEJMra032424 15917386

[4] Rendic S , Carlo FJD . Human cytochrome P450 enzymes: a status report summarizing their reactions, substrates, inducers, and inhibitors. Drug Metab Rev. 1997;29(1‐2):413‐580. doi:10.3109/03602539709037591 9187528

[5] Ogu CC , Maxa JL . Drug interactions due to cytochrome P450. Bayl Univ Med Cent Proc Taylor & Francis. 2000;13(4):421‐423. doi:10.1080/08998280.2000.11927719 PMC1312247 16389357

[6] Backman JT , Olkkola KT , Neuvonen PJ . Rifampin drastically reduces plasma concentrations and effects of oral midazolam. Clin Pharmacol Ther. 1996;59(1):7‐13. doi:10.1016/S0009-9236(96)90018-1 8549036

[7] Saari TI , Laine K , Leino K , Valtonen M , Neuvonen PJ , Olkkola KT . Effect of voriconazole on the pharmacokinetics and pharmacodynamics of intravenous and oral midazolam. Clin Pharmacol Ther. 2006;79(4):362‐370. doi:10.1016/j.clpt.2005.12.305 16580904

[8] Liu Y , Zhou S , Wan Y , Wu A , Palmisano M . The impact of co‐administration of ketoconazole and rifampicin on the pharmacokinetics of apremilast in healthy volunteers. Br J Clin Pharmacol. 2014;78(5):1050‐1057. doi:10.1111/bcp.12448 24962564 PMC4238785

[9] Guo H , Liu C , Li J , et al. A mechanistic physiologically based pharmacokinetic‐enzyme turnover model involving both intestine and liver to predict CYP3A induction‐mediated drug–drug interactions. J Pharm Sci. 2013;102(8):2819‐2836. doi:10.1002/jps.23613 23760985

[10] Gertz M , Houston JB , Galetin A . Physiologically based pharmacokinetic modeling of intestinal first‐pass metabolism of CYP3A substrates with high intestinal extraction. Drug Metab Dispos American Society for Pharmacology and Experimental Therapeutics. 2011;39(9):1633‐1642. doi:10.1124/dmd.111.039248 21632965

[11] Ohno Y , Hisaka A , Ueno M , Suzuki H . General framework for the prediction of Oral drug interactions caused by CYP3A4 induction from in vivo information. Clin Pharmacokinet. 2008;47(10):669‐680. doi:10.2165/00003088-200847100-00004 18783297

[12] Tod M , Goutelle S , Clavel‐Grabit F , Nicolas G , Charpiat B . Quantitative prediction of cytochrome P450 (CYP) 2D6‐mediated drug interactions. Clin Pharmacokinet. 2011;50(8):519‐530. doi:10.2165/11592620-000000000-00000 21740075

[13] Castellan A‐C , Tod M , Gueyffier F , et al. Quantitative prediction of the impact of drug interactions and genetic polymorphisms on cytochrome P450 2C9 substrate exposure. Clin Pharmacokinet. 2013;52(3):199‐209. doi:10.1007/s40262-013-0031-3 23344982

[14] Goutelle S , Bourguignon L , Bleyzac N , Berry J , Clavel‐Grabit F , Tod M . In vivo quantitative prediction of the effect of gene polymorphisms and drug interactions on drug exposure for CYP2C19 substrates. AAPS J. 2013;15(2):415‐426. doi:10.1208/s12248-012-9431-9 23319287 PMC3675754

[15] Steelandt J , Jean‐Bart E , Goutelle S , Tod M . A prediction model of drug exposure in cirrhotic patients according to child–Pugh classification. Clin Pharmacokinet. 2015;54(12):1245‐1258. doi:10.1007/s40262-015-0288-9 26070946

[16] Gabriel L , Tod M , Goutelle S . Quantitative prediction of drug interactions caused by CYP1A2 inhibitors and inducers. Clin Pharmacokinet. 2016;55(8):977‐990. doi:10.1007/s40262-016-0371-x 26936044

[17] Quantitative prediction of drug drug interactions ‐ DDI‐predictor academic version. 2020 https://www.ddi-predictor.org/. Accessed 16 Dec 2020

[18] Tod M , Goutelle S , Bleyzac N , Bourguignon L . A generic model for quantitative prediction of interactions mediated by efflux transporters and cytochromes: application to P‐glycoprotein and cytochrome 3A4. Clin Pharmacokinet. 2019;58(4):503‐523. doi:10.1007/s40262-018-0711-0 30194612

[19] Bayesian statistical modelling. 2nd ed. Wiley. Wiley.com. https://www.wiley.com/en‐us/Bayesian+Statistical+Modelling%2C+2nd+Edition‐p‐9780470018750. Accessed 4 Aug 2022

[20] Alexander SPH , Christopoulos A , Davenport AP , et al. The concise guide to pharmacology 2021/22: G protein‐coupled receptors. Br J Pharmacol. 2021;178:S27‐S156. doi:10.1111/bph.15538 34529832

[21] Enzymes. In: IUPHAR/BPS guide to PHARMACOLOGY. https://www.guidetopharmacology.org/GRAC/FamilyDisplayForward?familyId=690. Accessed 4 Feb 2026

[22] Bartkowski RR , Goldberg ME , Larijani GE , Boerner T . Inhibition of alfentanil metabolism by erythromycin. Clin Pharmacol Ther. 1989;46(1):99‐102. doi:10.1038/clpt.1989.112 2501060

[23] Palkama VJ , Isohanni MH , Neuvonen PJ , Olkkola KT . The effect of intravenous and Oral fluconazole on the pharmacokinetics and pharmacodynamics of intravenous alfentanil. Anesth Analg. 1998;87(1):190‐194. doi:10.1213/00000539-199807000-00039 9661572

[24] Kharasch ED , Vangveravong S , Buck N , et al. Concurrent assessment of hepatic and intestinal cytochrome P450 3A activities using deuterated alfentanil. Clin Pharmacol Ther. 2011;89(4):562‐570. doi:10.1038/clpt.2010.313 21346758 PMC3584707

[25] Saari TI , Laine K , Leino K , Valtonen M , Neuvonen PJ , Olkkola KT . Voriconazole, but not terbinafine, markedly reduces alfentanil clearance and prolongs its half‐life. Clin Pharmacol Ther. 2006;80(5):502‐508. doi:10.1016/j.clpt.2006.07.008 17112806

[26] Ludwig E , Graber H , Székely É , Csiba A . Metabolic interactions of ciprofloxacin. Diagn Microbiol Infect Dis. 1990;13(2):135‐141. doi:10.1016/0732-8893(90)90096-E 2369810

[27] Wahlländer A , Paumgartner G . Effect of ketoconazole and terbinafine on the pharmacokinetics of caffeine in healthy volunteers. Eur J Clin Pharmacol. 1989;37(3):279‐283. doi:10.1007/BF00679784 2612543

[28] Hebert MF , Roberts JP , Prueksaritanont T , Benet LZ . Bioavailability of cyclosporine with concomitant rifampin administration is markedly less than predicted by hepatic enzyme induction. Clin Pharmacol Ther. 1992;52(5):453‐457. doi:10.1038/clpt.1992.171 1424418

[29] Walko CM , Combest AJ , Spasojevic I , et al. The effect of aprepitant and race on the pharmacokinetics of cyclophosphamide in breast cancer patients. Cancer Chemother Pharmacol. 2012;69(5):1189‐1196. doi:10.1007/s00280-011-1815-5 22245954

[30] Varis T , Kivistö KT , Backman JT , Neuvonen PJ . The cytochrome P450 3A4 inhibitor itraconazole markedly increases the plasma concentrations of dexamethasone and enhances its adrenal‐suppressant effect. Clin Pharmacol Ther. 2000;68(5):487‐494. doi:10.1067/mcp.2000.110772 11103751

[31] Locniskar A , Greenblatt DJ , Harmatz JS , Zinny MA , Shader RI . Interaction of diazepam with famotidine and cimetidine, two H2‐receptor antagonists. J Clin Pharmacol. 1986;26(4):299‐303. doi:10.1002/j.1552-4604.1986.tb03527.x 2871051

[32] Kamali F , Thomas SHL , Edwards C . The influence of steady‐state ciprofloxacin on the pharmacokinetics and pharmacodynamics of a single dose of diazepam in healthy volunteers. Eur J Clin Pharmacol. 1993;44(4):365‐367. doi:10.1007/BF00316474 8513847

[33] Andersson T , Cederberg C , Edvardsson G , Heggelund A , Lundborg P . Effect of omeprazole treatment on diazepam plasma levels in slow versus normal rapid metabolizers of omeprazole. Clin Pharmacol Ther. 1990;47(1):79‐85. doi:10.1038/clpt.1990.12 2104790

[34] Gardner MJ , Baris BA , Wilner KD , Preskorn SH . Effect of sertraline on the pharmacokinetics and protein binding of diazepam in healthy volunteers. Clin Pharmacokinet. 1997;32(Supplement 1):43‐49. doi:10.2165/00003088-199700321-00007 9068935

[35] Dhillon S , Richens A . Valproic acid and diazepam interaction in vivo. Br J Clin Pharmacol. 1982;13(4):553‐560. doi:10.1111/j.1365-2125.1982.tb01421.x 6802161 PMC1402061

[36] Greiner B , Eichelbaum M , Fritz P , et al. The role of intestinal P‐glycoprotein in the interaction of digoxin and rifampin. J Clin Invest. 1999;104(2):147‐153. doi:10.1172/JCI6663 10411543 PMC408477

[37] Goey AKL , Meijerman I , Rosing H , et al. The effect of St John's wort on the pharmacokinetics of docetaxel. Clin Pharmacokinet. 2014;53(1):103‐110. doi:10.1007/s40262-013-0102-5 24068654

[38] Lobo ED , Bergstrom RF , Reddy S , et al. In vitro and in vivo evaluations of cytochrome P450 1A2 interactions with duloxetine. Clin Pharmacokinet. 2008;47(3):191‐202. doi:10.2165/00003088-200847030-00005 18307373

[39] Lundahl J , Regårdh CG , Edgar B , Johnsson G . Effects of grapefruit juice ingestion – pharmacokinetics and haemodynamics of intravenously and orally administered felodipine in healthy men. Eur J Clin Pharmacol. 1997;52(2):139‐145. doi:10.1007/s002280050263 9174684

[40] Saari TI , Laine K , Neuvonen M , Neuvonen PJ , Olkkola KT . Effect of voriconazole and fluconazole on the pharmacokinetics of intravenous fentanyl. Eur J Clin Pharmacol. 2008;64(1):25‐30. doi:10.1007/s00228-007-0398-x 17987285

[41] Ziesenitz VC , König SK , Mahlke NS , Skopp G , Haefeli WE , Mikus G . Pharmacokinetic interaction of intravenous fentanyl with ketoconazole. J Clin Pharmacol. 2015;55(6):708‐717. doi:10.1002/jcph.469 25651378

[42] Olkkola KT , Palkama VJ , Neuvonen PJ . Ritonavir's role in reducing fentanyl clearance and prolonging its half‐life. Anesthesiology. 1999;91(3):681‐685. doi:10.1097/00000542-199909000-00020 10485779

[43] Ha HR , Candinas R , Stieger B , Meyer UA , Follath F . Interaction between amiodarone and lidocaine. J Cardiovasc Pharmacol. 1996;28(4):533‐539. doi:10.1097/00005344-199610000-00009 8891878

[44] Feely J , Wilkinson GR , McALLISTER CB , Wood AJJ . Increased toxicity and reduced clearance of lidocaine by cimetidine. Ann Intern Med. 1982;96(5):592‐594. doi:10.7326/0003-4819-96-5-592 7073151

[45] Isohanni MH , Ahonen J , Neuvonen PJ , Olkkola KT . Effect of ciprofloxacin on the pharmacokinetics of intravenous lidocaine. Eur J Anaesthesiol. 2005;22(10):795‐799. doi:10.1017/S0265021505001316 16211753

[46] Isohanni MH , Neuvonen PJ , Palkama VJ , Olkkola KT . Effect of erythromycin and itraconazole on the pharmacokinetics of intravenous lignocaine. Eur J Clin Pharmacol. 1998;54(7):561‐565. doi:10.1007/s002280050513 9832299

[47] Olkkola KT , Isohanni MH , Hamunen K , Neuvonen PJ . The effect of erythromycin and fluvoxamine on the pharmacokinetics of intravenous lidocaine. Anesth Analg. 2005;100(5):1352‐1356. doi:10.1213/01.ANE.0000148123.79437.F9 15845683

[48] Noble DW , Bannister J , Lamont M , Andersson T , Scott DB . The effect of oral omeprazole on the disposition of lignocaine. Anaesthesia. 1994;49(6):497‐500. doi:10.1111/j.1365-2044.1994.tb03519.x 8017592

[49] Ujhelyi MR , O'Rangers EA , Fan C , Kluger J , Pharand C , Chow MSS . The pharmacokinetic and pharmacodynamic interaction between propafenone and lidocaine. Clin Pharmacol Ther. 1993;53(1):38‐48. doi:10.1038/clpt.1993.7 8422740

[50] Robson R , Wing L , Miners J , Lillywhite K , Birkett D . The effect of ranitidine on the disposition of lignocaine. Br J Clin Pharmacol. 1985;20(2):170‐173. doi:10.1111/j.1365-2125.1985.tb05053.x 4041336 PMC1400683

[51] McCrea JB , Majumdar AK , Goldberg MR , et al. Effects of the neurokinin1 receptor antagonist aprepitant on the pharmacokinetics of dexamethasone and methylprednisolone. Clin Pharmacol Ther. 2003;74(1):17‐24. doi:10.1016/S0009-9236(03)00066-3 12844131

[52] Booker BM , Magee MH , Blum RA , Lates CD , Jusko WJ . Pharmacokinetic and pharmacodynamic interactions between diltiazem and methylprednisolone in healthy volunteers. Clin Pharmacol Ther. 2002;72(4):370‐382. doi:10.1067/mcp.2002.127944 12386639

[53] Varis T , Kivistö KT , Backman JT , Neuvonen PJ . Itraconazole decreases the clearance and enhances the effects of intravenously administered methylprednisolone in healthy volunteers. Pharmacol Toxicol. 1999;85(3):29‐32. doi:10.1111/j.1600-0773.1999.tb01059.x 10426160

[54] Glynn AM , Slaughter RL , Brass C , D'Ambrosio R , Jusko WJ . Effects of ketoconazole on methylprednisolone pharmacokinetics and cortisol secretion. Clin Pharmacol Ther. 1986;39(6):654‐659. doi:10.1038/clpt.1986.114 3709030

[55] Brockmeyer NH , Breithaupt H , Ferdinand W , von Hattingberg M , Ohnhaus EE . Kinetics of oral and intravenous mexiletine: lack of effect of cimetidine and ranitidine. Eur J Clin Pharmacol. 1989;36(4):375‐378. doi:10.1007/BF00558298 2737230

[56] Majumdar AK , Yan KX , Selverian DV , et al. Effect of aprepitant on the pharmacokinetics of intravenous midazolam. J Clin Pharmacol. 2007;47(6):744‐750. doi:10.1177/0091270007300807 17463213

[57] Zhang X , Quinney SK , Gorski JC , Jones DR , Hall SD . Semiphysiologically based pharmacokinetic models for the inhibition of midazolam clearance by diltiazem and its major metabolite. Drug Metab Dispos American Society for Pharmacology and Experimental Therapeutics. 2009;37(8):1587‐1597. doi:10.1124/dmd.109.026658 19420129

[58] Tsunoda SM , Velez RL , von Moltke LL , Greenblatt DJ . Differentiation of intestinal and hepatic cytochrome P450 3A activity with use of midazolam as an in vivo probe: effect of ketoconazole. Clin Pharmacol Ther. 1999;66(5):461‐471. doi:10.1016/S0009-9236(99)70009-3 10579473

[59] Krishna G , Moton A , Ma L , et al. Effects of oral posaconazole on the pharmacokinetic properties of oral and intravenous midazolam: a phase I, randomized, open‐label, crossover study in healthy volunteers. Clin Ther. 2009;31(2):286‐298. doi:10.1016/j.clinthera.2009.02.022 19302901

[60] Gorski JC , Vannaprasaht S , Hamman MA , et al. The effect of age, sex, and rifampin administration on intestinal and hepatic cytochrome P450 3A activity. Clin Pharmacol Ther. 2003;74(3):275‐287. doi:10.1016/S0009-9236(03)00187-5 12966371

[61] Wang Z , Gorski JC , Hamman MA , Huang S‐M , Lesko LJ , Hall SD . The effects of St John's wort (Hypericum perforatum) on human cytochrome P450 activity. Clin Pharmacol Ther. 2001;70(4):317‐326. doi:10.1016/S0009-9236(01)00127-8 11673747

[62] Drug approval package: Ketek (telithromycin) NDA #021144. https://www.accessdata.fda.gov/drugsatfda_docs/nda/2004/21-144_Ketek.cfm. Accessed 3 Aug 2021

[63] Teng R , Butler K . The effect of ticagrelor on the metabolism of midazolam in healthy volunteers. Clin Ther Elsevier. 2013;35(7):1025‐1037. doi:10.1016/j.clinthera.2013.06.003 23870610

[64] Schoerlin M‐P , Mayersohn M , Hoevels B , Eggers H , Dellenbach M , Pfefen J‐P . Cimetidine alters the disposition kinetics of the monoamine oxidase‐a inhibitor moclobemide. Clin Pharmacol Ther. 1991;49(1):32‐38. doi:10.1038/clpt.1991.6 1988238

[65] Holtbecker N , Fromm MF , Kroemer HK , Ohnhaus EE , Heidemann H . The nifedipine‐rifampin interaction. Evidence for induction of gut wall metabolism. Drug Metab Dispos American Society for Pharmacology and Experimental Therapeutics. 1996;24(10):1121‐1123. doi:10.1016/s0090-9556(25)08415-6 8894514

[66] Harten JV , van Brummelen P , Lodewijks MT , Danhof M , Breimer DD . Pharmacokinetics and hemodynamic effects of nisoldipine and its interaction with cimetidine. Clin Pharmacol Ther. 1988;43:332‐341. doi:10.1038/clpt.1988.40 3345623

[67] Blum RA , Majumdar A , McCrea J , et al. Effects of aprepitant on the pharmacokinetics of ondansetron and granisetron in healthy subjects. Clin Ther. 2003;25(5):1407‐1419. doi:10.1016/S0149-2918(03)80128-5 12867217

[68] Villikka K , Kivistö KT , Neuvonen PJ . The effect of rifampin on the pharmacokinetics of oral and intravenous ondansetron. Clin Pharmacol Ther. 1999;65(4):377‐381. doi:10.1016/S0009-9236(99)70130-X 10223773

[69] Li Y , Sun D , Palmisano M , Zhou S . Slow drug delivery decreased total body clearance and altered bioavailability of immediate‐ and controlled‐release oxycodone formulations. Pharmacol Res Perspect. 2016;4(1):e00210. doi:10.1002/prp2.210 26977300 PMC4777261

[70] Nolan PE , Marcus FI , Hoyer GL , Bliss M , Gear K . Pharmacokinetic interaction between intravenous phenytoin and amiodarone in healthy volunteers. Clin Pharmacol Ther. 1989;46(1):43‐49. doi:10.1038/clpt.1989.104 2743707

[71] Zürcher RM , Frey BM , Frey FJ . Impact of ketoconazole on the metabolism of prednisolone. Clin Pharmacol Ther. 1989;45(4):366‐372. doi:10.1038/clpt.1989.42 2639662

[72] Twum‐Barima Y , Carruthers SG . Quinidine‐rifampin interaction. N Engl J Med. 1981;304(24):1466‐1469. doi:10.1056/NEJM198106113042405 7231477

[73] Arlander E , Ekström G , Alm C , et al. Metabolism of ropivacaine in humans is mediated by CYP1A2 and to a minor extent by CYP3A4: an interaction study with fluvoxamine and ketoconazole as in vivo inhibitors. Clin Pharmacol Ther. 1998;64(5):484‐491. doi:10.1016/S0009-9236(98)90131-X 9834040

[74] Hebert MF , Fisher RM , Marsh CL , Dressler D , Bekersky I . Effects of rifampin on tacrolimus pharmacokinetics in healthy volunteers. J Clin Pharmacol. 1999;39(1):91‐96. doi:10.1177/00912709922007499 9987705

[75] Nix DE , DeVito JM , Whitbread MA , Schentag JJ . Effect of multiple dose oral ciprofloxacin on the pharmacokinetics of theophylline and indocyanine green. J Antimicrob Chemother. 1987;19(2):263‐269. doi:10.1093/jac/19.2.263 3571046

[76] Nafziger AN , May JJ , Bertino JS . Inhibition of theophylline elimination by diltiazem therapy. J Clin Pharmacol. 1987;27(11):862‐865. doi:10.1002/j.1552-4604.1987.tb05580.x 3429693

[77] Paulsen O , Höglund P , Nilsson L‐G , Bengtsson H‐I . The interaction of erythromycin with theophylline. Eur J Clin Pharmacol. 1987;32(5):493‐498. doi:10.1007/BF00637676 3622597

[78] Nielsen‐Kudsk JE , Buhl JS , Johanuessen AC . Verapamil‐induced inhibition of theophylline elimination in healthy humans. Pharmacol Toxicol. 1990;66(2):101‐103. doi:10.1111/j.1600-0773.1990.tb00713.x 2315261

[79] Tremaine LM , Wilner KD , Preskorn SH . A study of the potential effect of sertraline on the pharmacokinetics and protein binding of tolbutamide. Clin Pharmacokinet. 1997;32(Supplement 1):31‐36. doi:10.2165/00003088-199700321-00005 9068933

[80] Ahonen J , Olkkola KT , Salmenpera M , Hynynen M , Neuvonen PJ . Effect of diltiazem on midazolam and alfentanil disposition in patients undergoing coronary artery bypass grafting. Anesthesiology. 1996;85(6):1246‐1252. doi:10.1097/00000542-199612000-00004 8968170

[81] Chiu LM , Menhinick AM , Johnson PW , Amsden GW . Pharmacokinetics of intravenous azithromycin and ceftriaxone when administered alone and concurrently to healthy volunteers. J Antimicrob Chemother. 2002;50(6):1075‐1079. doi:10.1093/jac/dkg003 12461037

[82] Bartlett NL , Lum BL , Fisher GA , et al. Phase I trial of doxorubicin with cyclosporine as a modulator of multidrug resistance. J Clin Oncol. 1994;12(4):835‐842. doi:10.1200/JCO.1994.12.4.835 8151326

[83] Rushing DA , Raber SR , Rodvold KA , Piscitelli SC , Plank GS , Tewksbury DA . The effects of cyclosporine on the pharmacokinetics of doxorubicin in patients with small cell lung cancer. Cancer. 1994;74(3):834‐841. doi:10.1002/1097-0142(19940801)74:3<834::AID-CNCR2820740308>3.0.CO;2-9 8039111

[84] Bisogno G , Cowie F , Boddy A , Thomas HD , Dick G , Pinkerton CR . High‐dose cyclosporin with etoposide ‐ toxicity and pharmacokinetic interaction in children with solid tumours. Br J Cancer. 1998;77(12):2304‐2309. doi:10.1038/bjc.1998.383 9649150 PMC2150390

[85] Lum BL , Kaubisch S , Yahanda AM , et al. Alteration of etoposide pharmacokinetics and pharmacodynamics by cyclosporine in a phase I trial to modulate multidrug resistance. J Clin Oncol. 1992;10:1635‐1642. doi:10.1200/JCO.1992.10.10.1635 1403041

[86] Mojtahedzadeh M , Sadray S , Hadjibabaie M , Fasihi M , Rezaee S . Determination of theophylline clearance after cimetidine infusion in critically ill patients. J Infus Nurs. 2003;26(4):234‐238. doi:10.1097/00129804-200307000-00008 12869856

[87] Gutfeld MB , Welage LS , Walawander CA , Wilton JH , Harrison NJ . The influence of intravenous cimetidine dosage regimens on the disposition of theophylline. J Clin Pharmacol. 1989;29(7):665‐669. doi:10.1002/j.1552-4604.1989.tb03398.x 2760260

[88] Gaska JA , Tietze KJ , Rocci ML , Vlasses PH . Theophylline pharmacokinetics: effect of continuous versus intermittent cimetidine IV infusion. J Clin Pharmacol. 1991;31(7):668‐672. doi:10.1002/j.1552-4604.1991.tb03754.x 1894763

[89] Fromm MF , Kauffmann H‐M , Fritz P , et al. The effect of rifampin treatment on intestinal expression of human MRP transporters. Am J Pathol. 2000;157(5):1575‐1580. doi:10.1016/S0002-9440(10)64794-3 11073816 PMC1885746

[90] Glaeser H , Drescher S , Eichelbaum M , Fromm MF . Influence of rifampicin on the expression and function of human intestinal cytochrome P450 enzymes. Br J Clin Pharmacol. 2005;59(2):199‐206. doi:10.1111/j.1365-2125.2004.02265.x 15676042 PMC1884745

[91] Watkins PB , Wrighton SA , Schuetz EG , Molowa DT , Guzelian PS . Identification of glucocorticoid‐inducible cytochromes P‐450 in the intestinal mucosa of rats and man. J Clin Invest. 1987;80(4):1029‐1036. doi:10.1172/JCI113156 3654968 PMC442342

[92] Wacher VJ , Silverman JA , Zhang Y , Benet LZ . Role of P‐glycoprotein and cytochrome P450 3A in limiting oral absorption of peptides and peptidomimetics. J Pharm Sci. 1998;87(11):1322‐1330. doi:10.1021/js980082d 9811484

[93] Asaumi R , Nunoya K , Yamaura Y , Taskar KS , Sugiyama Y . Robust physiologically based pharmacokinetic model of rifampicin for predicting drug–drug interactions via P‐glycoprotein induction and inhibition in the intestine, liver, and kidney. CPT Pharmacometrics Syst Pharmacol. 2022;11(7):919‐933. doi:10.1002/psp4.12807 35570332 PMC9286720

[94] Qian C , Zhao K , Chen Y , Liu L , Liu X . Simultaneously predict pharmacokinetic interaction of rifampicin with oral versus intravenous substrates of cytochrome P450 3A/P‐glycoprotein to healthy human using a semi‐physiologically based pharmacokinetic model involving both enzyme and transporter turnover. Eur J Pharm Sci. 2019;134:194‐204. doi:10.1016/j.ejps.2019.04.026 31047967

