# Supplementary material for: Quantitative prediction of intravenous drug interactions caused by cytochromes P450 inhibitors and inducers
Source: Br J Clin Pharmacol. 2026 Apr 4;92(8):2682–92. doi: 10.1002/bcp.70548 (PMC13421067; doi:10.1002/bcp.70548)
Supplement: Supplementary file 1 — Table S1. Induction Potency and Inhibition Ratio of all perpetrators used during our study. All values are given for oral intake. IX: induction or inhibition ratio. The inhibition ratio refers to values between −1 and 0, while the induction ratio refers to values greater than 0. [file BCP-92-2682-s001.docx]

Supplementary Material S 1

Table 1: Induction Potency and Inhibition Ratio of all perpetrators used during our study. All values are given for oral intake. *IX: induction or inhibition ratio. The inhibition ratio refers to values between −1 and 0, while the induction ratio refers to values greater than 0.*

| Precipitant | IX3A4 | IX2D6 | IX2C9 | IX2C19 | IX1A2 |
| --- | --- | --- | --- | --- | --- |
| amiodarone | -0.42 | -0.6 | -0.4 | 0 | 0 |
| aprepitant | -0.77 | 0 | 0.48 | 0 | 0 |
| cimetidine | -0.44 | -0.2 | 0 | 0 | -0.4 |
| ciprofloxacine | 0 | 0 | 0 | 0 | -0.37 |
| diltiazem | -0.8 | 0 | 0 | 0 | 0 |
| erythromycin | -0.82 | 0 | 0 | 0 | 0 |
| fluconazole | -0.85 | 0 | -0.65 | -0.78 | 0 |
| fluvoxamine | -0.3 | -0.36 | 0 | -0.98 | -0.99 |
| Grapefruit juice | -0,51 | 0 | 0 | -0,5 | 0 |
| itraconazole | -0.95 | 0 | 0 | 0 | 0 |
| ketoconazole | -0.98 | 0 | -0.49 | 0 | 0 |
| omeprazole | -0.28 | 0 | 0 | -0.43 | 0 |
| posaconazole | -0.74 | 0 | 0 | 0 | 0 |
| propafenone | 0 | -0.99 | 0 | 0 | -0.43 |
| ranitidine | -0.37 | 0 | 0 | 0 | 0 |
| rifampicin | 7,7 | 0 | 1,22 | 4,2 | 1,44 |
| ritonavir | -0.96 | -0.79 | 1.12 | 8.17 | 2.21 |
| sertraline | 0 | -0.41 | 0 | 0 | 0 |
| St Jonh's wort | 1,2 | 0 | 0,9 | 0,71 | 0 |
| telithromycin | -0.91 | 0 | 0 | 0 | 0 |
| terbinafine | 0 | -0.92 | 0 | 0 | -0.23 |
| ticagrelor | -0,35 | 0 | 0 | 0 | 0 |
| valproic acid | 0 | 0 | -0.61 | 0 | 0 |
| verapamil | -0.71 | 0 | 0 | 0 | 0 |
| voriconazole | -0.98 | 0 | -0.66 | -0.64 | 0 |

Exemple regarding equation 3 and 4:

We considered an interaction with a single cytochrome for clarity, such as the midazolam and telithromycin combination. Telithromycin is known to have an inhibition ratio (IR) of 0.91 on CYP3A4, and the AUC ratio $({AUC}^{*}/AUC)$ following intravenous administration of midazolam is 2.15, as reported in the study. Using the proposed equation,

$CR=\frac{(\frac{{AUC}^{*}}{AUC})-1}{\left( \frac{{AUC}^{*}}{AUC} \right)*IR}$ (eq. 3)

$$CR=\frac{(\frac{159}{73.8})-1}{\left( \frac{159}{73.8} \right)*0.91}=0.58$$

The intravenous contribution ratio (CR) introduced into our model is therefore 0.58 prior to re-estimation by Bayesian simulation. This value is incorporated into the Bayesian estimation, with all other CYP values set to a prior of 0, as midazolam is metabolized exclusively by CYP3A4. The Bayesian procedure then estimates the intravenous contribution ratio for $IV {CR}_{CYP 3A4}$ based on all previously calculated priors.

In our example, the estimated $IV {CR}_{CYP 3A4}$ for midazolam was 0.75 after Bayesian estimation, whereas it was 0.91 following oral administration.
